# Tall stature and gigantism in transition age: clinical and genetic aspects—a literature review and recommendations

**DOI:** 10.1007/s40618-023-02223-z

**Published:** 2023-10-27

**Authors:** V. Sada, G. Puliani, T. Feola, R. Pirchio, R. Pofi, F. Sesti, D. De Alcubierre, M. E. Amodeo, F. D’Aniello, L. Vincenzi, D. Gianfrilli, A. M. Isidori, A. B. Grossman, E. Sbardella, the TALENT group, A. M. Savage, C. Foresta, C. Krausz, C. Durante, M. C. De Martino, D. Paoli, R. Ferrigno, S. Caiulo, M. Minnetti, V. Hasenmajer, C. Pozza, G. Kanakis, B. Cangiano, M. Tenuta, F. Carlomagno, A Di Nisio, F. Pallotti, M. G. Tarsitano, M. Spaziani, F. Cargnelutti, I. Sabovic, G. Grani, C. Virili, A. Cozzolino, I. Stramazzo, T. Filardi

**Affiliations:** 1 Department of Experimental Medicine, Sapienza University of Rome, Viale Regina Elena 324, 00161 Rome, Italy; 2 Oncological Endocrinology Unit, IRCCS Regina Elena National Cancer Institute, Rome, Italy; 3 Neuroendocrinology, Neuromed Institute, IRCCS, Pozzilli, Italy; 4 Dipartimento di Medicina Clinica e Chirurgia, Sezione di Endocrinologia, Università Degli Studi di Napoli “Federico II”, Naples, Italy; 5 Oxford Centre for Diabetes, Endocrinology, and Metabolism, Churchill Hospital, Oxford University Hospitals, NHS Trust, Oxford, UK; 6 Dipartimento Pediatrico Universitario Ospedaliero, Bambino Gesù Children Hospital, Rome, Italy; 7 Centre for Rare Diseases (ENDO-ERN Accredited), Policlinico Umberto I, Rome, Italy; 8 Green Templeton College, University of Oxford, Oxford, UK; 9 Centre for Endocrinology, Barts and the London School of Medicine, London, UK

**Keywords:** Gigantism, Tall stature, Transition age, GH

## Abstract

**Purpose:**

Tall stature is defined as height greater than the threshold of more than 2 standard deviations above the average population height for age, sex, and ethnicity. Many studies have described the main aspects of this condition during puberty, but an analysis of the characteristics that the physician should consider in the differential diagnosis of gigantism—tall stature secondary to a pituitary tumour—during the transition age (15–25 years) is still lacking.

**Methods:**

A comprehensive search of English-language original articles was conducted in the MEDLINE database (December 2021-March 2022). We selected all studies regarding epidemiology, genetic aspects, and the diagnosis of tall stature and gigantism during the transition age.

**Results:**

Generally, referrals for tall stature are not as frequent as expected because most cases are familial and are usually unreported by parents and patients to endocrinologists. For this reason, lacking such experience of tall stature, familiarity with many rarer overgrowth syndromes is essential. In the transition age, it is important but challenging to distinguish adolescents with high constitutional stature from those with gigantism. Pituitary gigantism is a rare disease in the transition age, but its systemic complications are very relevant for future health. Endocrine evaluation is crucial for identifying conditions that require hormonal treatment so that they can be treated early to improve the quality of life and prevent comorbidities of individual patient in this age range.

**Conclusion:**

The aim of our review is to provide a practical clinical approach to recognise adolescents, potentially affected by gigantism, as early as possible.

## Introduction

Adult height shows distinct variability in the general population, following a normal Gaussian distribution dependent on age, sex, ethnicity, as well as many other factors. Human growth leading to final height is a composite and dynamic process, associated with phenotypic changes in stature, body proportions, and composition, reflecting the interplay of genetic, environmental, hormonal, nutritional, and socio-economic factors [1].

Different endocrine factors regulate growth during each period of life, determining separate but closely integrated phases whereby many hormones influence transient growth and final height [2]. During the intrauterine phase, fetal growth is critically dependent on insulin and Insulin-like Growth Factors (IGF), both maternal and placental, and nutritional status [3]. In the early years of life, nutrition is a pivotal factor, while in childhood, a crucial role is played by the GH/IGF1 axis. Nevertheless, thyroid hormones, adrenal androgens, glucocorticoids, sex steroids, ghrelin, leptin, and insulin are all known to participate in the growth process through their interactions with the GH/IGF1 axis. During puberty, the growth spurt depends on the interaction between the somatotroph and gonadal axes, which act synergistically towards the achievement of final stature [4, 5].

In a clinical context, deviations from a normal growth pattern can often represent the first evidence of a huge spectrum of diseases, encompassing both endocrine and non-endocrine disorders [6, 7]. While these deviations often manifest as growth inhibition or delay, excessive growth leading to tall stature may also reflect underlying pathological alterations.

Traditionally, ‘tall stature’ in children is defined as a height exceeding the 97.7^th^ percentile or two standard deviations (SD) above the mean height for a population of the same age, sex, and ethnicity [8]. As a result, 2.3% of children fall into the category of tall stature and thus may be considered worthy of investigation [9]. Furthermore, children presenting with height within the normal range, but with a projected height surpassing 2SD above their mid-parental height, may also be evaluated. Although the percentage of children with tall stature is equal to that of children with ‘short stature’, referrals to paediatric endocrinologists for the assessment of tall stature appear to be far less frequent than those for short stature. This is probably due to a better societal acceptance of tall stature, along with the assumption that increased height in a child with tall parents is not alarming and is simply an acceptable familial trait. Indeed, clinical referrals become more likely whenever height exceeds > 2.5SD or > 3SD (extremely tall stature), respectively, 0.6% and 0.1% of the population [1]. Nevertheless, such patients require intensive investigation to establish any underlying pathological cause of increased growth, and to address potential problems of social adaptation [10].

‘Familial tall stature’, also known as constitutional tall stature, represents the most common cause and is considered as a variant of the normal pattern of childhood growth and development [11]. However, despite their rarity, many pathologic conditions also present with tall stature and may be associated with severe comorbidities [12]. Therefore, differentiating between healthy tall children and those with underlying diseases, while ruling out chromosomal, genetic, and endocrine disorders in the latter [13], poses a significant clinical challenge.

In the assessment of such children, measurement of current height, growth velocity, weight, head circumference, and body mass index (BMI) should be evaluated [14]. Additional diagnostic information should be gathered from birth data (weight, length, and head circumference), family history (height and pubertal timing of both parents), developmental history, and growth curve review, if available [15]. Assessment of body proportions is also critical: specifically, a head circumference >  + 2SD associated with tall stature could help clinicians to identify overgrowth syndromes, such as the Beckwith-Wiedemann, Sotos, Perlman, Simpson–Golabi–Behmel, Tatton–Brown–Rahman and Weaver syndromes [16, 17]. These overgrowth syndromes typically affect childhood from the prenatal to the postnatal phases, involving the development of the patients, in particular Beckwith-Wiedemann and Sotos syndromes that may be associated with hormone imbalance and increased susceptibility to malignancy [17]. The presence of cardiovascular abnormalities, skin anomalies, skeletal malformations, facial dysmorphisms, abnormalities of the genitalia, and neurodevelopmental delay, may also suggest an underlying syndromic cause [18].

However, many of these genetic conditions exhibit overlapping phenotypes, thus complicating the differential diagnosis, particularly when dealing with patients in transition or adult age. Similar to children with short stature, ‘constitutionally tall’ individuals referred to the endocrinologist for an evaluation of the GH/IGF1 axis typically show no clear biochemical abnormalities [19]. Indeed, ‘pituitary gigantism’, excessive stature due to a primary hypothalamic-pituitary abnormality, is an extremely rare disease, with an estimated annual incidence of 8 per million, with only several hundred cases reported to date [20]. It may be a sporadic and isolated condition, and approximately half of the cases occur within the context of a concurrent hereditary syndrome or follow a familial inheritance pattern [3]. Excessive GH levels not only cause dramatic linear growth acceleration but can also lead to mild to moderate obesity, progressive macrocephaly, prognathism, and changes in glucose metabolism, including type 2 diabetes [21]. It is therefore vital, given the relevant number of comorbidities connected to a prolonged diagnostic delay, to identify such patients early and initiate appropriate therapy [22].

Although most children with short or tall stature do not have an underlying pathological condition, extreme deviations from average height, especially beyond + 3SDs, require further investigation. This review aims to provide a practical clinical approach to identify patients in the transition age (15–25 years) who may have underlying hypothalamic-pituitary defects, as opposed to idiopathic/constitutional tall stature. We conducted a comprehensive search of English-language original articles in the MEDLINE (PubMed) database between December 2021 and March 2022. The search used free text words in combination with Medical Subject Heading (MeSH) terms. The keywords applied for the search included the “*Gigantism*” term as a keyword and “*human*” as a filter.

## Clinical approach

### Auxology with a focus on the transition age

As noted above, tall stature is generally defined as height measurements exceeding the threshold >  + 2SDs above the average population  height for age, sex, and ethnicity, corresponding to the 97^th^ percentile of each growth chart [14]. Ideally, each country should have its own growth chart. In this regard, Natale et al. performed a systematic review comparing data from the World Health Organization (WHO) *Multicenter Growth Reference Study* with data from studies performed in 55 countries or ethnic groups, including over 11 million children from economically advantaged backgrounds. They highlighted differences in average stature between groups, identifying a ‘tall group’ (with height means three or more ages above the + 0.5SD mark compared to the general population) in Europeans and Pacific Islanders, thus suggesting that the use of a single international standard for anthropometric measurements may not be entirely justified. As a result, they created a large-scale comparison of growth in healthy children around the world [23]. In case a country-specific growth chart is unavailable, patients should nevertheless be referred to WHO growth charts for children and adolescents, spanning from birth to 19 years of age. According to the WHO, a height at + 2SD in adults corresponds to 191.1 cm in men and 176.2 cm in women (WHO, 2006) [24].

Tall stature is also defined by a height value over >  + 2SDs above the Target Height (TH) SD score. TH can be derived by calculating the mean height of both parents and then adding or subtracting 6.5 cm for boys and girls, respectively. The definition of the target range, considering the overall secular trend of increasing average height, typically falls within 1.6–2 SD of the TH-SDs. This definition helps clinicians to identify cases of familial idiopathic tall stature, especially in the absence of dysmorphism or known parental disease [6].

#### Puberty

When interpreting tall stature, several factors should be taken into account, including age, sex, genetics, nutrition, and pubertal development [25, 26]; notably, the latter is one of the most relevant elements, especially during the transition age. A correct evaluation of sexual development should include pubertal assessment according to Tanner's staging system, evaluating genital and breast changes, as well as the development of pubic and axillary hair in both girls and boys, while for the measurements of testicular volume in boys, the Prader orchidometer is useful [27]. Reassuring indicators suggesting a diagnosis of non-pathological tall stature include: tall stature accompanied by normal pubertal progression; height within the TH associated with regular Height Velocity (HV), and bone age corresponding to chronological age [28]. A recent study showed that girls with tall stature may enter puberty earlier but remain in the normal range for pubertal onset, compared with girls with normal or short stature, probably influenced by IGF1 levels [29]. Conversely, when the auxological parameters do not develop harmonically – especially in the case of altered pubertal progression—various pathological scenarios should be considered.

Focusing on the transition age, when evaluating a child for tall stature, one condition to be excluded is precocious puberty, when the child can initially present with temporarily increased growth due to the anabolic effect of sex steroids; however, it should be noted that this condition ultimately leads to a *short* final height if left untreated, due to the gonadal steroid-induced premature closure of the epiphyses [30–32]. Another aspect that should be considered is overweight/obesity, which has been widely described as a risk factor for central precocious puberty [33], especially in girls. For this reason, when evaluating a tall child with overweight/obesity, careful physical examination of sexual development should be carried out to rule out any signs of pubertal onset. Other forms of precocious puberty associated with tall stature may be part of syndromic conditions, such as the McCune–Albright syndrome (MAS) [34] or Neurofibromatosis Type 1 (NF1) [35]. On the contrary, extremely delayed puberty in tall adolescents could be indicative of gonadal failure, as seen in Klinefelter Syndrome [36].

### Pituitary gigantism

Since gigantism is caused by GH/IGF1 excess which occurs before the fusion of the epiphyseal growth plates, it is a condition exclusively observed in children and adolescents, either before or during puberty. The transition age is a critical phase, since elevated levels of serum GH and IGF1 can cause rapid, excessive linear growth, potentially resulting in extremely tall adult stature if left unchecked. Mild to moderate obesity commonly accompanies tall stature in these patients; specifically, in children with GH excess, the abnormal height growth typically precedes or occurs simultaneously with rapid weight gain [37], whereas children with exogenous obesity typically exhibit increases in their weight percentile before any changes in height are noted [38]. In contrast, in adulthood, because of complete epiphyseal fusion, GH excess has no effect on stature and is responsible for the clinical features of acromegaly [37, 39].

Dramatic linear growth acceleration usually prompts initial medical investigation in children. The mean onset of rapid growth in children with pituitary gigantism has been reported to occur at the age of 13 years, and even earlier in girls [39]. Among different forms of gigantism, X-linked acro-gigantism (XLAG) is associated with the earliest onset of rapid growth, with a median age of onset of 1.5 years; these patients typically reach their final height at 23.5 years, deviating from the TH by about 10.9 cm (6.52%). Conversely, individuals with *AIP* mutations and those with genetically negative forms of gigantism typically show a later onset of growth acceleration (13–14 years), reaching their final height at 19–20 years with a more significant difference from TH, approximately 19–21 cm (10.9–12.7%) [39].

## Clinical presentation of gigantism during the transition age

Gigantism and acromegaly represent two clinical manifestations of the same pathological entity—namely a GH-secreting pituitary adenoma, also known as a pituitary neuroendocrine tumour (PitNET). The clinical phenotype largely depends on the timing of disease onset in relation to skeletal maturation. GH excess determines a continuum of clinical manifestations that can occur both before and after the fusion of the epiphyseal growth plates, with frequent overlap [40]: many of these patients will have features of acromegaly in conjunction with gigantism, hence the term ‘acro-gigantism’.

Apart from scattered case reports, only two studies have reported the clinical presentation of patients with pituitary gigantism diagnosed during the transition age [39, 41, 42]: a single-centre study by Colao et al. conducted on 13 patients diagnosed between 15 and 20 years, and a multi-centre study by Rostomyan et al. investigating more than 200 patients with a wider age at diagnosis (median 21 years, interquartile range 15.5–27) [39]. Tall stature is usually the first clinical sign that leads to medical attention, thus initiating the diagnostic process for gigantism. In particular, patients with pituitary gigantism show a peculiar growth pattern, in which the young patient, who was not initially born large for gestational age, progressively crosses higher percentiles during childhood, eventually reaching an adult height above 2SD and surpassing their genetic TH [9]. The onset of growth acceleration has been demonstrated to occur significantly earlier in females than in males (median age of onset: 11 *vs* 13 years). Additionally, a shorter diagnostic delay from symptoms onset to diagnosis has been found in females, resulting in a significantly lower age at the time of gigantism in females than in males (median age at diagnosis: 15.8 *vs* 21.5 years) [39]. Therefore, males are more likely to be diagnosed during the transition age. Of note, not all patients reportedly had attained their final height at the time of diagnosis, particularly male patients [39]. While tall stature is generally the primary presentation-presenting feature, the pathological effects of the prolonged exposition to supraphysiological levels of GH and IGF1 are systemic. Patients with gigantism may also show acral enlargement and facial changes, which represent the second most frequent clinical sign (37%). The median shoe size reported at diagnosis was 48 (EU) in males and 42 in females. Acromegalic features were already present at diagnosis in patients with gigantism regardless of sex and age, although facial changes were less commonly observed in patients aged < 19 years [39]; similarly, signs and symptoms typical of acromegaly such as joint disorders and sweating were rarely encountered in younger patients.

Since most patients with gigantism often harbour macro- and giant pituitary tumours, signs and symptoms of compression are frequently observed at diagnosis, with headache and visual field defects being reported in 23% and 12% of patients, respectively. In addition to visual field impairment, lachrymation, transitory eyelid palsy, or ptosis have also been reported [41]. Furthermore, around a quarter of patients exhibited at least one pituitary deficit; hypogonadism was diagnosed in 40% of patients at diagnosis [39]. In line with these findings, one study reported the presence of amenorrhoea, both primary and secondary, in all female patients with gigantism [41]. Prolactin co-secretion has been reported in more than 30% of cases, particularly in patients with invasive and extrasellar pituitary tumours, with galactorrhoea reportedly being slightly more frequent in females [39]. Moreover, typical GH excess complications such as sleep apnoea, carpal tunnel syndrome, hypertension, and glucose metabolism disorders were already present at diagnosis, particularly in patients aged > 20 years [39].

Alterations in glucose and lipid metabolism may be seen in patients with gigantism; insulin-resistance has been mainly found at diagnosis [41], whereas glucose intolerance and overt diabetes mellitus have been reported less frequently at diagnosis, particularly in patients < 19 years [39].

Concerning cardiovascular disease, cardiac impairment was detected at diagnosis in 36.5% of cases, primarily involving left ventricular hypertrophy (21%) and diastolic dysfunction (10%) [39]. In one study comparing the echocardiographic parameters of six males diagnosed with pituitary gigantism during adulthood with those of six age- and sex-matched acromegalic patients and ten healthy controls, both groups of patients displayed significantly higher left ventricular mass index, interventricular septum diastolic thickness, and posterior wall thickness compared to controls. Although patients with gigantism exhibited a significantly longer disease duration, no relevant differences in cardiac structure and performance were noted in these patients when compared to acromegalic patients. However, individuals with cardiac abnormalities in the gigantism group exhibited higher IGF1 levels than those with a normal cardiac structure. For this reason, the authors of this paper suggest performing echocardiography regardless of disease duration to detect cardiac impairment early [40]. Apart from one case [40], no alterations in blood pressure or heart rate have been found in patients with gigantism [41].

Thus, in patients diagnosed during the transition age, it is necessary to not only focus on tall stature and external changes but also investigate potential systemic complications.

## Clinical approach

The clinical approach to tall children during transition age should include, wherever possible:

- *Birth data (weight, length, and head circumference*);
- *Familial auxological parameters: height/weight for parents and first-degree relatives, pubertal timing of parents (age of menarche of the mother, age of the pubertal growth spurt of the father);*
- *Personal medical history: hypo/hyper-glycaemia, metabolic disorders, over-feeding, cardiac defects, ocular defects, anosmia, ligamentous laxity, joint dislocation, obesity, and neurodevelopmental disorders;*
- *Assessment of standing height, sitting height, arm span, weight, BMI and head circumference, as compared to country-specific growth charts;*
- *Assessment of pubertal status according to Prader’s scale, Marshall and Tanner staging;*
- *Assessment of HV: calculated at least every 6 months, expressed in cm per year (cm/yr) with particular attention to peak-height-velocity indicating a pubertal spurt *[43]*;*
- *Clinical evaluation: cardiac murmurs, anomalies of the skin, skeletal examination (pectus excavatum, scoliosis), and facial dysmorphism;*

The initial approach to a tall child in the transition age should also incorporate the determination of bone age according to a standardised model (for example, the Greulich and Pyle atlas or the Tanner-Whitehouse atlas version 2 or 3 [44]) to distinguish between a physiological constitutional growth delay or familial tall stature—characterised by a normal/delayed bone age—and pathological precocious puberty, characterised by advanced bone age. Especially during the peri-pubertal transition age, it is crucial that growth assessments be performed regularly; when determining the normality of a child's growth pattern, serial height measurements of HV calculations are more useful than a single height-for-age percentile. A child that grows regularly on a high percentile (even above the 97^th^ percentile), without significant comorbidities, and especially with a family history of tall stature, should generally be considered a normal variant. Conversely, rapid acceleration of growth, regardless of the percentile, should be investigated further to rule out pathological causes [45]. Transient tall stature can also be observed in patients with true precocious and pseudo-precocious puberty.

The most prevalent cause of tall stature is familial tall stature, characterised by tall parents, normal growth velocity, normal findings on physical examination, and correspondence between bone age and the chronologic age. Stature generally remains in the target genetic range [8]. Sometimes this condition is characterised by an acceleration in growth velocity in early childhood, between 2 and 4 years of age. Growth progression remains slightly above the normal curve, following the same centiles until puberty. Rarely, children may also exhibit advanced bone age and early pubertal development within the normal range. Pagani et al*.* suggested the possibility of GH hypersecretion in children with familial tall stature, as supported by the presence of age- and sex-adjusted IGF1 levels in the upper range of normal, or hypersensitivity to GH [46].

Another physiological cause of tall stature is, paradoxically, constitutional delay of growth and development (CDGD). This may occur not only in children from short or normal-statured families but also in children of tall-stature families. A study published in 2005 analysed a cohort of adolescents aged 12–16 years and demonstrated that the final height of CDGD children exceeded the mean TH by more than 4 cm, reaching a mean value of + 1.9 and 2.1SD for boys and girls, respectively—consistent with final tall stature—in 42% of cases [47].

Certain genetic conditions, such as Marfan Syndrome, may be characterised by tall stature, with a rapid increase in growth velocity occurring just before or in the early stages of the transition age. For this reason, Disease-specific Growth Charts of Marfan Syndrome patients have been developed in some countries. The syndrome, caused by mutations in the fibrillin-1 (*FBN1,* chromosome 15q) gene and dysregulation of transforming growth factor β (TGFβ), affects the skeletal system, resulting in tall stature, abnormally long and slender limbs, fingers, and toes, chest wall abnormality, and scoliosis. The arm span is greater than their height, with an arm span-to-height ratio greater than 1, while the upper/lower segment ratio is diminished [48]. A Korean study showed that the 50th percentile of height in patients with Marfan Syndrome exceeds the normative 97^th^ percentile for both genders [49]. A French study comparing more than 250 Marfan patients to a control population demonstrated that Marfan children’s overgrowth decreases with age, especially during the transition phase, at about 17 years of age [50]. Another important aspect deals with the specific mutation of Marfan Syndrome since patients carrying *TGFBR2* mutations have lower mean height than patients harbouring *FBN1* mutations [51].

Klinefelter syndrome also exhibits its peculiar growth pattern, with normal auxological parameters during infancy, followed by a rapid growth tall stature between 5 and 8 years of age, and further growth in the pubertal period [52]. Thus, disease-specific growth charts can be useful for monitoring growth patterns, planning the timing of growth-reductive therapy if necessary, and predicting adult height.

In conclusion, for correct identification of tall stature, it is necessary to report the height value on country-specific or disease-specific growth charts. A systematic clinical approach, along with the periodic monitoring of auxological parameters and HV, is essential for distinguishing between physiological and pathological causes of tall stature.

Table 1 summarises the main differential diagnoses for tall stature.

**Table 1:** Main differential diagnoses for tall stature

Differential diagnosis	Bone age	Centile crossing	HVR	Puberty (N, P or D)	Anatomical features
Transition Age					
Familial Tall Stature	↑/ →	< 2 SD	→	N	Normal appearance
FIPA	NA	> 2 SD	NA	N	Tall stature
MAS	↑	NA	↑	P	Café-au-lait spots, skeletal lesions (fibrous osteodysplasia), craniofacial dysplasia (optic and auditory nerve impairment)
Marfan syndrome	NA	> 97th percentile	↑	N	Abnormally long and slender limbs, fingers, toes, chest wall, and scoliosis
Klinefelter syndrome	→	> 2 SD	↑/ → ^*^	N	Small, firm testes; gynecomastia; high-pitched voice; learning disability
Hyperthyroidism	↑	< 2 SD	↑	N	Goiter, tachycardia, hypertension, diarrhea, exophthalmos
Obesity	↑	> 2 SD	↑	P	BMI > 95th percentile
Childhood					
Beckwith-Wiedemann syndrome	↑	≥ 2 SD	↓^*^	NA	Macroglossia, abdominal wall defects, congenital heart disease,
Sotos syndrome	↑	> 2 SD	↑	P^#^	Macrodolichocephaly, facial alteration
Weaver syndrome	↑	> 98th percentile	NA	NA	Abnormal facial alteration
Simpson–Golabi–Behmel syndrome	↑	> 97th percentile	NA	NA	Macrocephaly, ocular hypertelorism (wide-spaced eyes) with broad upturned nose, macroglossia, and macrostomia (large mouth), supernumerary nipples, pectus excavatum, and hypotonia
Perlman syndrome	NA°	75th–97th percentile	NA°	NA°	Macrosomia, macrocephaly, round facies, hypotonia, visceromegaly, cryptorchidism and inguinal hernia
Tatton–Brown–Rahman syndrome	NA	N/ > 2 SD	NA	P	Macrocephaly noticed at birth, joint hyperlaxity, scoliosis, hypotonia, and seizures

*HVR* Height Velocity Rate; *SD* Standard Deviation; *N* normal onset, *P* precocious, *D* Delayed, *NA* Not Available; *FIPA* Familial Isolated Pituitary Adenomas; *NF1* Neurofibromatosis type 1; *MAS* McCune-Albright Syndrome; *BMI* Body Mass Index

^#^especially in female patients

^*^after 8 years old, before HVR is increased

°due to high mortality rate in the neonatal period

## Genetics

### Genetic background of acro-gigantism

**Fig. 1:**
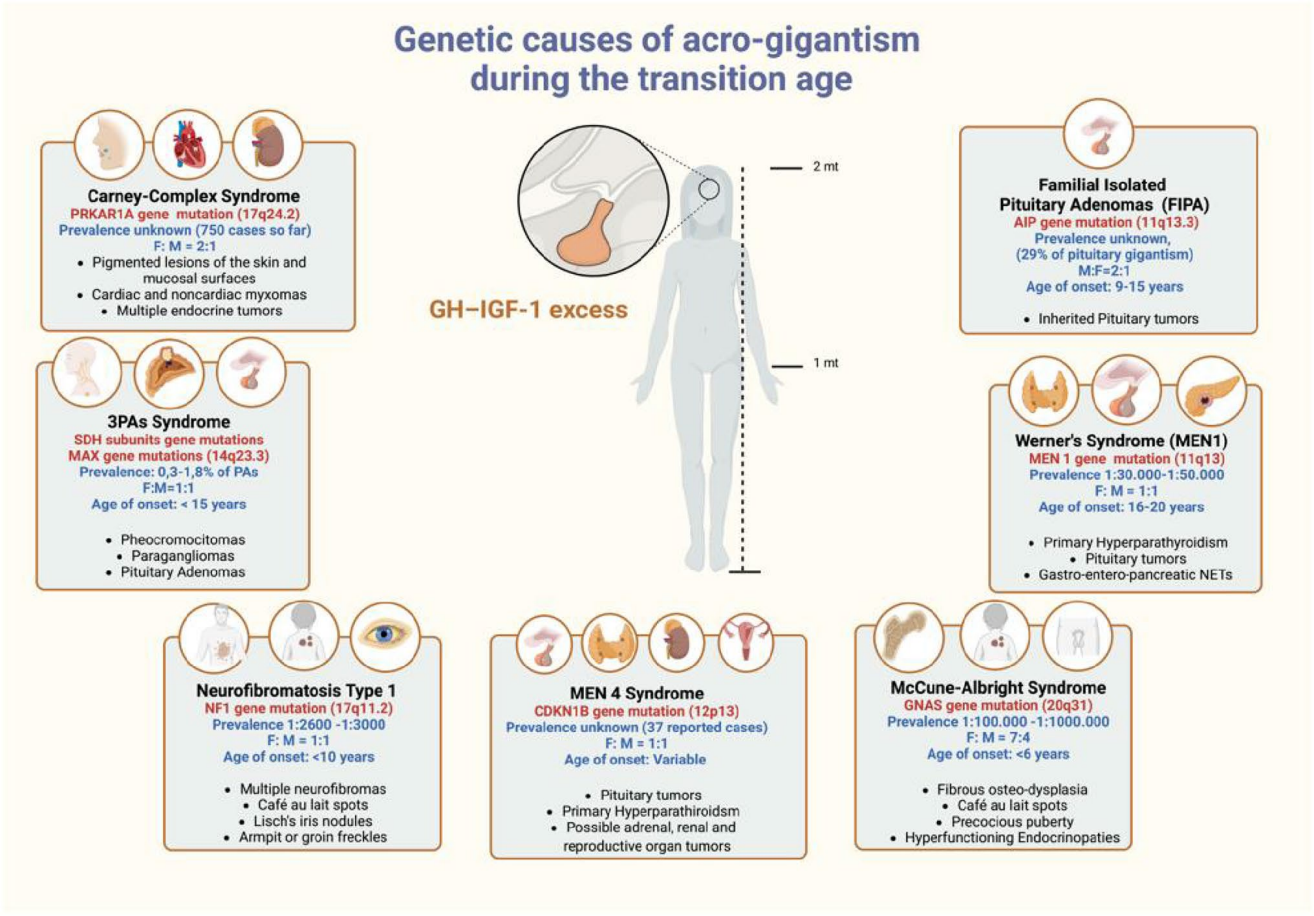
Representation of possible genetic causes of acro-gigantism during the transition age

Although the most frequent pathological cause of GH excess is represented by apparently sporadic PitNETs, around half of all patients with gigantism have a genetic background. In adolescence and young adulthood, the most common genetic causes of acro-gigantism are *familial isolated pituitary adenomas* (FIPA), in which pituitary tumours occur in two or more family members without other syndromic manifestations, and multiple neuroendocrine neoplasia type 1 (MEN1), in which pituitary tumours are associated (often not synchronously) with primary hyperparathyroidism (pHPT), or other NETs, generally of gastro-enteropancreatic origin (GEP-NETs) [53]. Exceptionally, acro-gigantism can also manifest in adolescent patients affected by multiple neuroendocrine neoplasia type 4 (MEN4) or other rare genetic syndromes such as the Carney complex or MAS. However, the putative genetic cause of acro-gigantism remains unclear in approximately 50% of patients and often associated with more aggressive tumour behaviour [39]. Figure 1 represents the main genetic causes of acro-gigantism in the transition age.

### Familial isolated pituitary adenoma (FIPA)

In the transition age, the most frequent cause of acro-gigantism in patients with FIPA is the presence of loss-of-function mutations in the gene coding for the aryl hydrocarbon receptor-interacting protein (AIP) [54], which account for 29% of cases of pituitary gigantism, as reported in the largest multicentre European study to date (208 patients with gigantism) [39]. *AIP* is a tumour suppressor gene, located on chromosome 11q13.3, encoding for a co-chaperone protein involved in the signalling of cyclic adenosine monophosphate through the binding with phosphodiesterase subtype 4A5, although it has been associated with many additional functions. Mutations of *AIP* are inherited in an autosomal dominant manner but with an incomplete, generally low (around 30%) penetrance, and high phenotypic variability [55, 56]. These genetic characteristics may explain why germline *AIP* mutations can also be found in patients with apparent sporadically diagnosed GH-secreting tumours, even in the absence of family history, most likely reflecting the low penetrance rather than the onset of de novo mutations [57]. Genetic diagnosis is based on gene sequencing and, if negative, on multiple ligation probe amplification (MLPA) [53].

Patients with a germline *AIP* mutation typically present large, invasive tumours (macroadenomas 90%, and giant 10% [39]) often with an extrasellar extension, a higher likelihood of pituitary apoplexy, and frequently aggressive clinical behaviour [58]. The typical age of onset is in the second decade of life, with symptoms manifesting before 18–19 years in most cases (65–71.4%) [39, 58]. Gigantism is observed in about a third of cases [55]; typical features of acromegaly are also common despite the young age of onset, depending on GH/IGF1 levels and the diagnostic delay [39]. Indeed, rapid growth acceleration typically starts at a median age of 13 years (9–15 years), with a delay from first symptoms to diagnosis of 3 years (1–6 years) [39], which contributes to a longer period of linear growth that may also be exacerbated by concomitant hypogonadism [39]. A male predominance has been observed in most of the published series [39, 59–62], unlike other causes of gigantism that are more common in females (X-LAG, MEN1, unknown genetic causes). Of note, early diagnosis in *AIP* mutation carriers leads to the detection of smaller lesions with less suprasellar extension or cavernous sinus invasion, which are therefore less difficult to manage compared to clinically symptomatic cases [58]. Similarly, earlier diagnosis, with associated accelerated disease control, may also help to reduce the final height in such patients [39], further demonstrating the benefits of genetic and clinical screening for pituitary diseases in carrier patients via genetic counselling and surveillance. When the *AIP* mutation is found in the proband, genetic counselling should be proposed to all first-degree relatives, as the disease may already manifest as early as the age of 4 years [63]. Regular clinical follow-up should be performed in *AIP* mutations carriers throughout the transition age, with an annual physical examination and hormone assessment (GH, IGF1, and prolactin) and baseline MRI, followed by 5-yearly scans, until the age of 20; in case no abnormalities are detected, the clinical follow-up in the second decade of life can be less frequent [58].

Following the identification of a loss-of-function variant (p. Arg703Gln) in the PAM (peptidylglycine α-amidating mono-oxygenase) gene in a three-member FIPA acro-gigantism family, a recent study by Trivellin et al*.* examined 299 individuals with sporadic pituitary adenomas and 17 FIPAs kindreds, identifying rare PAM variants in two subjects with sporadic acromegaly and gigantism (p.Gly552Arg and p.Phe759Ser, respectively), suggesting that PAM could be a candidate gene associated with their disease. These patients varied from micro- to macroadenomas. Further studies, including more subjects affected by the most common pituitary hypersecretion such as hyperprolactinemia, are needed to better explain the possible role of PAM in pituitary tumorigenesis [64].

### Multiple endocrine neoplasia type 1 (MEN1) and type 4 (MEN4)

MEN1 is an autosomal dominant disorder predisposing to the development of neoplasms, mostly in neuroendocrine tissues [65]. It is caused by inactivating mutations of the *MEN1* gene, located on 11q13, which encodes for menin, a protein involved in cell proliferation, histone methylation, and gene transcription [66, 67]. The prevalence of MEN1 is currently estimated to be between 1/30,000 and 1/500,000 [68, 69]. From a clinical standpoint, the syndrome can occur in a sporadic (10% of cases) or a familial setting (90% of cases), usually within the fourth decade of life [70]. MEN1 is characterised by high penetrance, with 95% of mutation carriers showing biochemical evidence of disease and 80% developing clinical signs by the age of 50 years [71]. PitNETs are the first MEN1-associated lesion in 25% of sporadic and 10% of familial cases [72], with a subtype distribution reflecting the one observed in the general population. Moreover, MEN1-PitNETs have been described as more frequent in female patients, as well as being larger, more invasive lesions, less controllable by standard treatments [73], although this has been disputed.

**Fig. 2:**
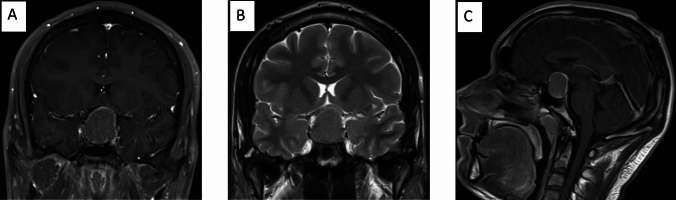
GH-secreting macroadenoma in 21-year-old patient. **A** and **B** illustrate coronal T1-weighted post-contrast and T2-weighted MRI images at diagnosis showing a large homogeneous pituitary mass with intra and suprasellar extension, compression, and upward stretching of the optic chiasm. In addition, there is a cavernous sinus extension, internal carotid artery compression. **C** shows sagittal T1-weighted MRI images with the presence of a large homogeneous pituitary mass with partial hyper attenuated margins

Despite the high penetrance of MEN1, data regarding paediatric and adolescent patients are scarce, as only a few studies have retrospectively reported data regarding clinical outcomes and natural history in young MEN1 patients [25, 74–77]. In this regard, a large study by Goudet et al. [75] retrospectively analysed 122 MEN1 patients under 21 years, describing their clinical symptoms, and biological and/or imaging abnormalities. The presence of a pituitary lesion was seen in 34% of these young patients, mainly in the 16–20 years age group. Interestingly, no PitNETs occurred before the age of 10 years; moreover, in the entire cohort, only 2% of pituitary lesions turned out to be GH-secreting. Similarly, in several retrospective cohorts focusing on young MEN1 patients, no somatotrophinomas were reported, confirming these to be extremely rare in adolescent MEN1 patients and even rarer in the paediatric age [25, 74–77]. As a result, pituitary gigantism is an uncommon finding in young MEN1 patients; the largest series to date, which analysed 208 patients with gigantism across multiple European centres, reported identifiable MEN1 mutations in just 1% of patients [39].

It should be noted that, in MEN1 patients, GH excess and subsequent acromegaly may instead derive from ectopic growth hormone releasing-hormone (GHRH) secretion by neuroendocrine pancreatic tumours [78]. Interestingly, MEN1 has been reported to account for up to 76% of GHRH-secreting NETs [79]. In this context, diagnosis usually occurs during the third decade of life [78] with a slight female predominance (60% of cases) [79].

The presentation of gigantism due to ectopic GHRH secretion, generally from a well-differentiated NET [78, 80], is characterised by accelerated growth with abnormal height without mass-related symptoms (headache and/or visual impairment); notably, GH excess reverts after surgical resection of the NET [80].

In 2006, germline mutations in the cyclin-dependent kinase inhibitor 1b gene (*CDKN1B*), encoding for the known oncosuppressor p27, were detected in patients exhibiting MEN1-like features with no apparent *MEN1* mutation [81]. This finding led to the identification of MEN4, an extremely rare autosomal dominant condition, which has only been described in a handful of case reports worldwide to date [82]. Patients with MEN4 present with a MEN1-like phenotype and are therefore prone to the development of NETs, including PitNETs [83] in up to one-third of total cases [82]. Specifically, MEN4-related somatotrophinomas have been reported to occur in all age groups [81, 84, 85]; however, due to the rarity of the disease, data regarding paediatric and/or transition-age patients are still lacking, with a single case reported to date. Sambugaro et al. [85] reported on a patient with early onset, aggressive acromegaly in a 30-year-old woman. At age 5, the patient had undergone clinical investigations due to excessive growth velocity, leading to the diagnosis of GH hypersecretion due to a pituitary macroadenoma. Despite multimodal treatment, biochemical control was not achieved, requiring long-term medical therapy. Genetic investigations did not detect germline *AIP* or *MEN1* mutations, leading to the identification of a deletion in the *CDKN1B* 5′-UTR region and the diagnosis of MEN4 [85].

### Rare genetic syndromes

Carney complex syndrome (CCS) is a rare genetic syndrome including multiple endocrine and non-endocrine neoplasms, whose diagnosis is based on the presence of two or more of the following manifestations: skin pigmentation, cardiac myxomas, primary pigmented nodular adrenocortical lesions, large cell calcifying Sertoli cell tumours, ductal adenomas, pustular melanomas, blue naevi, osteochondral myxomas, thyroid tumours, and acromegaly [86]. In 30% of cases, it is caused by de novo mutations in the suppressor gene for PRKAR1A (*CNC1*) at 17q24.2, encoding for the regulatory subunit type 1α of the protein kinase A [87]. CCS is generally diagnosed over 20 years of age; in this context, acromegaly is observed in 10–18% of cases, with a female predominance [86]. In a French multicentre prospective study including 70 CCS patients (50 females, mean age 35.4 ± 16.7 years, 81% carrying a PRKAR1A gene mutation), annual systematic screening highlighted that acromegaly had a prevalence of 18%, although clinical signs of GH excess were generally mild or absent. A higher percentage of patients (30%) presented with non-diagnostic biochemical abnormalities of the somatotroph axis [87]; interestingly, “subclinical acromegaly” might also be associated with the development of cardiac myxomas [87]. Other genetic alterations associated with CCS include abnormalities in *CNC2*, located at 2p16, and activating mutations for PRKAR1B, the catalytic subunit beta (Cβ) of PKA [86, 88]. The latter was found in a young woman with CCS who presented at 19 years old with acromegaly, pigmented spots, and a myxoma [88].

MAS is another rare cause of acro-gigantism during the transition age. This syndrome, caused by somatic gain-of-function mutations of the *GNAS* gene encoding the α-subunit of the Gs signalling protein, is characterised by the presence of skeletal lesions (fibrous osteodysplasia), café-au-lait spots, and hyperfunctioning endocrinopathies such as precocious puberty [89]. Acro-gigantism in MAS typically affects male patients, with a variable age of onset – ranging from childhood to young adulthood—and frequent prolactin co-secretion. GH excess may worsen the skeletal deformities associated with this syndrome, especially craniofacial dysplasia, resulting in optic and auditory nerve impairment [86], and complicates pituitary surgery.

Over the last decade, new genetic causes of multiple endocrine tumours have been identified. Co-existing phaeochromocytomas and pituitary adenomas (3Pas) are usually associated with SDHx mutations, in which pituitary tumours are larger and more aggressive, generally occurring in adulthood, although several cases have been described in young patients. Specifically, 9 cases of GH-secreting tumours (one co-secreting prolactin), all macro-tumours, have been reported in patients younger than 30 years [90]. Recently, the Liege group described three cases of phaeochromocytomas associated with pituitary adenomas in patients with germline heterozygous *MAX* exon deletions. Two of these cases showed a GH-secreting tumour occurring at a relatively young age (a 26-year-old female and a 16-year-old male) [91].

Lastly, GH excess during the transition age and young adulthood may be associated with NF1 syndrome. In a recently published retrospective case series, two cases of young patients exhibiting clinical and biochemical acromegaly, a 14-year-old Hispanic male with a macroadenoma and a 29-year-old Caucasian female with a pituitary hyperplasia, were reported [92].

In conclusion, during the transition age the presence of *AIP* mutations should be suspected in patients with acro-gigantism, particularly in males, with an onset in adolescence (< 18y) or young adulthood (20-30y), independent of a positive family history. Conversely, gigantism is an extremely rare occurrence in the context of MEN1 and MEN4, and then with a female preponderance; rarely, acro-gigantism may be diagnosed in adolescence and young adulthood in the context of other genetic disorders, such as CCS, MAS, 3Pas, MAX-associated tumours, NF1, and the recently identified PAM variants.

Early diagnosis and treatment have been demonstrated to halt clinical progression and prevent further growth, and therefore genetic counselling in this age group is mandatory.

## Other differential diagnoses

### Hyperthyroidism

Hyperthyroidism is uncommon during childhood and adolescence, with an annual incidence in childhood ranging from 1 to 6.5 per 10,000 individuals in different studies [93–95]. The most frequent cause is Graves’ disease; other differential diagnosis includes MAS, activating mutations of the TSH receptor gene, toxic nodules, and exogenous thyroid hormone administration [96]. Linear growth can also be affected, resulting in increased HV rate and advanced bone age [97]. Interestingly, the final height is generally not compromised, with only some patients achieving a final height exceeding the estimated target [97]. However, more frequently, children are below 2SD for height [98]. In addition, the appropriate treatment strategy determines the adult final height within the normal range [9]. Thyroid hormones have been shown to increase the expression and release of GH from the pituitary in animal studies [99]. Conversely, patients affected by hypothyroidism show lower levels of IGF1 [100, 101], and treatment with levothyroxine has been found to increase serum levels of IGFBP1 [102]. Thyroid hormones also modulate the biological effects of GH and IGF1 on target tissues [103]. Therefore, it is essential to assess thyroid function in all patients presenting with tall stature.

#### Obesity

Many studies have demonstrated that obesity in children is associated with an increase in growth velocity and final stature. In fact, children affected by obesity have been reported to be 4–5 cm taller than normal-weight controls, with an advanced bone age and early puberty [9, 104]. In this context, one study investigating potential differences in stature and skeletal maturity, in 521 subjects from birth to 18 years according to BMI, demonstrated that obesity was associated with increased final height, especially for girls aged 10–12 years, and for boys aged 11–13 years. Moreover, in overweight/obese adults, skeletal maturity is advanced throughout childhood [105]. The impact of obesity on final height seems to vary depending on the individual’s age of onset and sex: obesity at approximately 11 years of age was associated with the most increase in height (5.7 cm in females and 4.5 cm in males) [106].

Obesity causes a decrease in GH secretion and a blunted response of GH to various stimuli [107], probably due to increased somatostatin levels [108]. On the contrary, IGF1 levels are usually normal or high, presumably because of the effects of insulin. One study demonstrated that obesity could increase IGF1 levels in pre-pubertal children, who have a greater response to GH compared to children with tall stature [109]. Concurrently, circulating levels of ghrelin are lower in obese children and adolescents compared to their normal-weight peers, with circulating levels correlating with the degree of insulin resistance [110]. Thus, obesity can result in an increase in growth velocity, especially if the condition appears in the late pre-pubertal or early pubertal phase, and should therefore be considered in the differential diagnosis of tall stature.

#### Doping

The term ‘doping’ refers to the use of performance-enhancing substances in non-pharmacologic doses to improve sports performance [111]. Abuse of GH is widespread and has been reported in 27% of young male weightlifters [112], even extending beyond professional contexts [113]. During childhood and adolescence, abuse of GH causes further damage, since the achievement of normal pubertal growth and adult body composition is dependent on the GH/IGF1 and hypothalamo-pituitary–gonadal axes, but data are scarce [114, 115]. During adolescence and the transition age, detecting GH abuse can be challenging, since GH and IGF1 levels naturally rise in this period, but the *GH-2000* method, which also assesses pro-collagen type III N-terminal peptide levels, also seems to be a reliable detection tool in this age group. [116].

## Biochemical diagnosis

### GH and IGF1 assessment

The differential diagnosis of tall stature is complex given the heterogenous presentation of the rare conditions that cause pathological increases in height [39, 117, 118]. There are currently no evidence-based recommendations to determine which patients should be evaluated for pathological causes of tall stature, or to inform the best strategy for investigation. A thorough clinical evaluation is mandatory before going through the biochemical assessment [119].

Generally, serum IGF1 is recommended as the best screening test due to its excellent linear dose–response correlation with 24 h mean GH secretion [120]. Nevertheless, no published studies have ever defined a dedicated IGF1 reference range to guide the difficult differential diagnosis between tall stature and gigantism. It is, therefore, crucial to use age-referenced ranges, as potential misdiagnoses may arise when evaluating normal adolescents due to their significantly increased IGF1 levels during puberty with respect to adulthood [121, 122]. A study by Creo et al., focusing on patients with pituitary gigantism, showed that the subjects’ IGF1 levels did not significantly differ much from the normal laboratory range, thus demonstrating that relying solely on IGF1 values may not suffice for diagnosing gigantism, highlighting the need for an integrated diagnostic approach including clinical features and growth patterns [123]. Furthermore, recent evidence has highlighted a potential role of the IGF2 protein, traditionally known for its involvement in normal fetal development due to its secretion via the placenta during pregnancy. In the post-natal period, IGF2 secretion mostly derives from hepatocytes independent of GH secretion. Interestingly, IGF2 overexpression has been observed in two rare conditions characterized by fetal overgrowth, namely Beckwith-Wiedemann and Perlman syndrome [17, 124]; however, its potential role in acro-gigantism still requires elucidation.

Random/morning serum GH levels interpretation is also challenging, being affected by its pulsatility, lack of uniform assay standardisation, poor reproducibility between laboratories, and, importantly, the lack of reliable reference values for sensitive immunometric assays [125]. Some authors have demonstrated basal GH levels to correlate with GH day-curves and nadir GH levels after an oral glucose tolerance test (OGTT) [126, 127], but these procedures are often neglected, being both time-consuming and cumbersome.

Nevertheless, pituitary adenomas in patients with gigantism are often highly secretory, leading to significantly elevated GH and IGF1 levels [128]. An OGTT test for GH levels associated with cranial MRI in children with height above 2SD and IGF1 circulating levels approaching the upper limit of normal are crucial steps in confirming or ruling out the diagnosis of gigantism [14].

Previous, and older, expert consensus suggested a failure to suppress serum GH levels to less than 5 μg/L after a 1.75 mg/kg oral glucose challenge (maximum, 75 g) as the gold standard for diagnosing gigantism [128]. However, there is no mention of a different diagnostic approach between acromegaly and gigantism in the latest *Endocrine Society* guidelines [129], therefore suggesting a serum GH cut-off of < 1 μg/L (or < 0.4 μg/L in ultra-sensitive new available assays [130]) within 2 h after a 75 g of oral glucose load to be regarded as confirmatory for gigantism [129]. Prolactin may be raised with pituitary tumours, while thyroid hormones, oestrogens, and androgens need assessment [3].

Notably, androgens mediate the growth spurt, partly through conversion to oestrogen but also through direct effects in the growth plate [2]. Children with precocious puberty or virilising disorders generally exhibit increased growth velocity and height SDs compared to their peers, which leads to advanced bone age and short stature in adulthood if not recognised, prevented, and treated [118, 131]. Conversely, conditions characterised by decreased levels or reduced sensitivity to sex hormones (hypogonadism, aromatase deficiency, and oestrogen resistance) can lead to prolonged growth due to delayed growth plate fusion. Although the growth rate in these cases is slow, these children keep growing into adulthood, developing tall stature (often with eunuchoid proportions) only later in life [2]. It is important to underline that treatment for pituitary gigantism (i.e., surgery and/or radiotherapy [132]) might lead to secondary hypogonadism [133], which may further increase the adult height if not adequately treated [39].

In cases of precocious puberty, further evaluation is often necessary. For patients showing a phenotype consistent with androgen effects, the most useful initial tests should include measurements of total testosterone, dehydroepiandrosterone sulphate, and 17-hydroxyprogesterone [134]. If oestrogen effects are more pronounced instead, screening tests should include LH and oestradiol for girls, and LH, β-human chorionic gonadotrophin, and oestradiol for boys. FSH levels have more limited usefulness in differentiating children with GnRH-dependent precocious puberty from non-progressive variants [135]. All these measurements should be performed in the morning using high-sensitivity assays designed for paediatric patients.

In patients with clinical sexual precocity and basal prepubertal LH, the gold standard for differentiating precocious puberty is the assessment of gonadotrophins (mainly LH) following stimulation with GnRH agonists [135, 136], which helps in establishing the level of activation of the gonadotroph axis. While several protocols have been used thus far [137–139], but an LH peak higher than 5 IU/L is indicative of an activated gonadotroph axis [140]. Baseline random LH measured through ultra-sensitive assay has been suggested as useful to assess the activation of the gonadotrophic axis, avoiding the need for GnRH testing [141]. However, data are not consistent, with a wide range of diagnostic sensitivity (from 60 to 100%) and cut-off values (ranging from 0.1 to 1.5 IU/L) [140, 142, 143]. Basal and GnRH-stimulated FSH concentrations do not seem to be helpful [135, 141], although suppressed GnRH-stimulated FSH concentrations suggest peripheral precocious puberty.

In contrast to girls, where low oestradiol concentrations do not rule out the diagnosis of precocious puberty [144, 145], in boys testosterone is an excellent marker for sexual precocity, because prepubertal concentrations of this hormone effectively excludes the diagnosis of precocious puberty [135].

## Pituitary magnetic resonance imaging (MRI)

MRI is the gold standard for the evaluation of the pituitary gland in the paediatric and transition-age population [146–148], providing morphological information and allowing the evaluation of size, signal characteristics, and vascularisation [148]. The adenohypophysis is isointense to grey matter on non-contrast T1 and T2-weighted standard Spin Echo sequences [149], whereas the neurohypophysis is characteristically hyperintense on T1 and hypointense on T2 sequences [149]. An appropriate imaging protocol should include sagittal and coronal T1-weighted and T2-weighted sequences, as well as contrast-enhanced T1- weighted images following intravenous injection of gadolinium [150]: normally, the pituitary gland enhances after gadolinium administration [148, 149]. Due to the small dimensions of the sellar structures and potential intrinsic lesions, acquiring small field-of-view images is essential [150], with either 2 or 3-mm sections obtained with 1.5 T scanning fields or 1.0 to 1.5-mm sections obtained with 3 T scanning[150].

Pituitary tumours have typically delayed enhancement and washout characteristics [148, 149]. Microadenomas are typically hypointense on both unenhanced and contrast-enhanced sequences, becoming iso/hyperintense to the normal pituitary gland in delayed sequences [148]. Conversely, macroadenomas are usually isointense in T1-weighted images and present intense contrast enhancement after gadolinium injection [148].

In adult patients with acromegaly, T2-hypointense adenomas are more common, smaller, and less invasive compared to T2-isointense and hyperintense tumours [151]. Moreover, patients with T2-hypointense adenomas also have higher IGF1 values at baseline [151, 152]. T2-weighted signal intensity is a marker for the granulation pattern [153, 154]; accordingly, T2-hypointense adenomas have been linked with better hormonal responses and greater tumour shrinkage after presurgical somatostatin analogue administration [151, 153, 155]. Currently, the few data reporting MRI findings in the paediatric and transition-age population mainly derive from retrospective studies. In the cohort of Rostmoyan et al*.,* the median age of rapid growth onset was 13 years (interquartile range 9–15), pituitary macroadenomas were more prevalent than microadenomas (84.3 vs 15.7%), with 15% of macroadenomas classified as ‘giant’ adenomas (> 4 cm); extrasellar extension was found in 89% of macroadenomas and extrasellar invasion in 64%. No differences were found between males and females [39]. In another study, Colao et al*.* reported data on the diagnosis and treatment of patients with GH-secreting adenomas with clinical onset in adolescence. Thirteen patients were enrolled, with a mean age of 17 ± 2 years; on MRI evaluation, the mean maximal tumour diameter was 21.8 ± 5.4 mm, and the mean tumour volume was 2756 ± 1895 mm^3^ [3, 41].

In conclusion, a pituitary MRI with an appropriate imaging protocol is mandatory for the evaluation of the pituitary gland in the paediatric and transition-age population with suspected gigantism (Fig. 2).

## Conclusions

Human growth is a complex process and discriminating healthy tall children from those affected by acro-gigantism due to underlying diseases, either related to genetic (FIPA, and MEN1, more rarely CCS, MAS, 3PAs, MAX-associated tumours, NF1, and the recent PAM variants), or endocrine alterations (hyperthyroidism, obesity), is a compelling challenge. In general, females tend to receive a diagnosis of gigantism at a younger age than males, therefore males are more likely to be diagnosed during the transition age. A thorough clinical evaluation, using country- and disease-specific growth charts, is crucial before the biochemical assessment with GH and IGF1 measurements. Currently, a dedicated IGF1 reference range to guide the difficult differential diagnosis between constitutional tall stature and gigantism is still lacking. Nevertheless, the pathological effects of the prolonged exposition to supraphysiological levels of GH and IGF1 can cause systemic complications, mainly metabolic and cardiovascular; therefore, the clinical evaluation of pubertal staging and other signs and symptoms is of most importance, especially during the transition age.

## Data Availability

No data or material to share.

## Code availability

No code to share.
